# Phagocytic Integrins: Activation and Signaling

**DOI:** 10.3389/fimmu.2020.00738

**Published:** 2020-04-30

**Authors:** Alvaro Torres-Gomez, Carlos Cabañas, Esther M. Lafuente

**Affiliations:** ^1^Department of Immunology, Ophthalmology and Otorhinolaryngology, School of Medicine, Universidad Complutense de Madrid, Madrid, Spain; ^2^Instituto de Investigación Sanitaria Hospital 12 de Octubre (i+12), Madrid, Spain; ^3^Severo Ochoa Center for Molecular Biology (CSIC-UAM), Madrid, Spain

**Keywords:** phagocytosis, integrins, signaling, CR3, Mac-1, complement

## Abstract

Phagocytic integrins are endowed with the ability to engulf and dispose of particles of different natures. Evolutionarily conserved from worms to humans, they are involved in pathogen elimination and apoptotic and tumoral cell clearance. Research in the field of integrin-mediated phagocytosis has shed light on the molecular events controlling integrin activation and their effector functions. However, there are still some aspects of the regulation of the phagocytic process that need to be clarified. Here, we have revised the molecular events controlling phagocytic integrin activation and the downstream signaling driving particle engulfment, and we have focused particularly on α_M_β_2_/CR3, α_X_β_2_/CR4, and a brief mention of α_V_β_5_/α_V_β_3_integrins.

## Introduction

Phagocytosis entails the engulfment and disposal of particles in sequential steps, including particle recognition, cytoskeletal remodeling, membrane protrusion, particle engulfment, and phagolysosomal digestion (1, 2). The role of integrins in phagocytosis is evolutionarily conserved and can be observed in *Caenorhabditis elegans* INA-1/PAT-3, which is involved in clearance of apoptotic cells (3), and *Drosophila* αPS3/βν, which has roles in microbial defense and apoptotic cell removal (4, 5) (Table 1). In mammals, the orthologues α_V_β_3_/α_V_β_5_ are expressed in professional and non-professional phagocytes (endothelial, epithelial, fibroblast, and neuronal and mesenchymal cells) with a role in phosphatidylserine-rich apoptotic/necrotic body clearance. Professional phagocytes in mammals express complement receptors α_M_β_2_*/*CR3 and α_X_β_2/_CR4, which are involved in host defense and tissue homeostasis (45). Other integrins with reduced phagocytic capacity (α_5_β_1_, α_2_β_1_, α_3_β_1_, and α_6_β_1_) are involved in phagocytosis of fibrillar or denatured extracellular matrix components (Table 1).

**Table 1:** Major mammalian phagocytic integrins and their invertebrate orthologues.

**Integrin**	**αI domain**	**Co-receptors**	**Phagocytic targets**	**Expression**
α_M_β_2_	+	- SR-A1/2 (6) - Dectin1 (7) - RAGE (8)	- iC3b-opsonized particles (9) - iC3b-opsonized particles (9) - C3d-opsonized particles (10) - Denatured proteins (11, 12) - Bacteria (LPS, LBP) (13, 14) - Zymosan (15, 16) - Myelin sheaths (17) - Platelet factor 4 (PF4) (18) - LL-37 (19)	Professional phagocytes
α_X_β_2_	+	–	- iC3b-opsonized particles (9) - Osteopontin (20) - Fibrillar α-synuclein (αSN) (21)	Professional phagocytes
α_2_β_1_	+	–	- Collagen fibrils (22–24)	Non-professional phagocytes
α_3_β_1_	–	- CD36/SCARB3 (25)	- Laminin (26)	Non-professional phagocytes
α_5_β_1_	–	–	- Fibronectin aggregates (27) - Fibronectin-opsonized apoptotic bodies (28) - Vitronectin (29)	Non-professional phagocytes
α_6_β_1_	–	- CD36/SCARB3 (25)	- Fibrillar β-amyloid (30, 31)	Professional phagocytes
α_V_β_3_	–	- TIM4 (32) - CD36/SCARB3 (33) - MerTK (34, 35)	- MFG-E8 opsonized (36, 37) - Gas6 through co-receptor (38) - ProS1 through co-receptor (39, 40) - TSP-1 (41)	Professional and non-professional phagocytes
α_V_β_5_	–		- Apoptotic or necrotic bodies (42, 43)	Professional and non-professional phagocytes
αPS3/βν	–	–	- Peptidoglycan (4, 44) - Apoptotic cells (4, 5)	*Drosophila* phagocytes.
INA-1/PAT-3	?	–	- Apoptotic cells (3)	*C. elegans* phagocytes

Integrins are characterized by requiring activation to be functional. This review has focused on the main events determining β_2_ integrin activation and downstream signaling in relation to cytoskeletal remodeling and particle engulfment, and it makes a special mention of the main differences between other phagocytic integrins, especially those involved in apoptotic cell clearance.

## Integrin Structure and Activation

**Figure 1:**
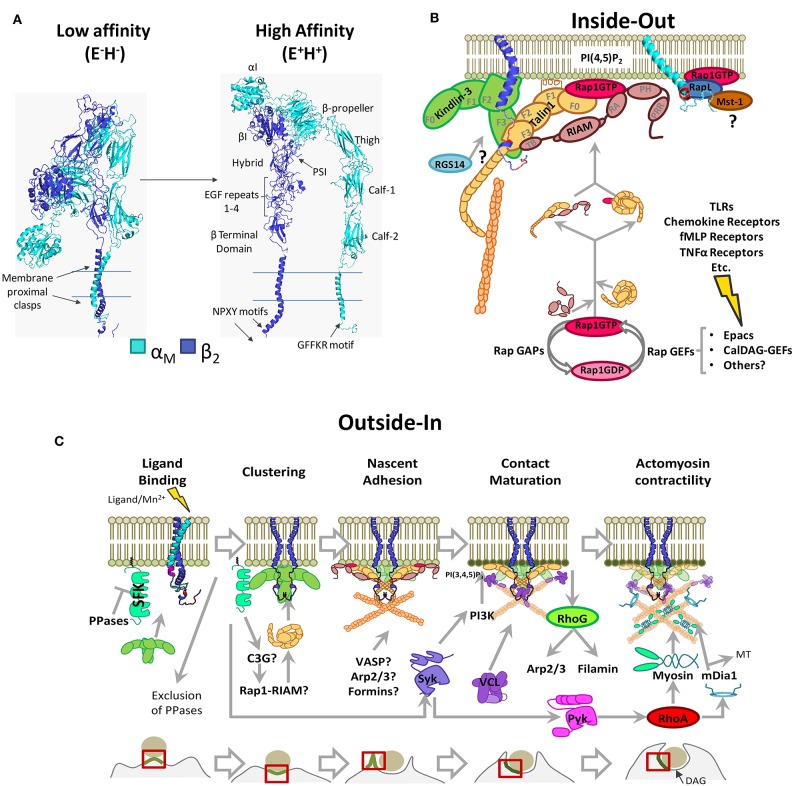
Phagocytic integrin α_M_β_2_ structure and activation pathways. **(A)** 3D structure model generated through homology modeling using Modeller 9.23. The following PBD entries served as templates: 1m8o, 2k9j, 2knc, and 3k6s (low-affinity/bent conformation), 1dpq, 2lkj, 2m3e, 2rn0, 2vdo, 3g9w, 3fcu, 5e6s, 6ckb, and 6avu (high affinity conformation), and the sequences for α_M_ (NP_001139280.1) and β_2_ (NP_000202.3). PSI: Plexin-Semaphorin-Integrin domain. **(B)** Inside-out pathway of integrin α_M_β_2_ activation. Signals stemming from multiple receptors induce Rap1-GTP loading and RIAM-mediated recruitment of Talin1 to integrin tails, with possible contributions by other pathways. Protein-binding motifs in the integrin tails are shown in red (NPXY) and in purple (GFFKR). FERM domains are highlighted for Kindlin-3 and Talin1 (F0–F3). Highlighted RIAM domains are as follows; TB, Talin1 Binding domain; RA, Rap Association domain; PH, Pleckstrin Homology domain; PRR, Prolin Rich Region. **(C)** Outside-In pathway in the context of phagocytosis through α_M_β_2_. Src Family Kinases remain inhibited by membrane-bound tyrosine phosphatases. Kindlin-3 mediated clustering facilitates Src Family Kinase activation, contact maturation and contractility necessary for phagocytic engulfment. PPases, Phosphatases; SFK, Src Family Kinases; MT, Microtubules. For simplicity, some proteins are shown as monomers. Question marks denote unsolved or hypothetical signaling steps.

Phagocytic integrins are heterodimeric (α and β subunit) receptors. Subunits are divided into ectodomains, a transmembrane helix, and short cytoplasmic tails. The α-subunit ectodomains contain Mg^2+^-binding metal-ion-dependent adhesive sites (MIDAS) and Adjacent to MIDAS (AdMIDAS), which binds inhibitory Ca^2+^ or activating Mn^2+^ (46, 47). Ligand binding can occur either at the αI-domain (α-subunit) in α_X_, α_M_, and α_2_ or at the α/β-chain interface in integrins without the αI domain (Figure 1A, Table 1).

Integrins are tightly regulated by conformational changes, a hallmark of which is cytoplasmic tail separation (48). Integrin conformations are described according to the state of the headpiece (open/closed; H^+^/H^−^) and leg ectodomains (extended/bent; E^+^/E^−^) (49). Resting integrins remain in an inactive/“bent” (E^−^H^−^) conformation with the lowest free energy (−4.0 kcal/mol for α_5_β_1_) with respect to fully activated integrins (50). E^−^H^−^is characterized by a closed ligand-binding site and clasped membrane proximal regions (51). In activated integrins (E^+^H^+^), the hybrid domain (β-subunit) swings away from the α-chain, and the membrane proximal regions unclasp. This correlates with the rearrangement of the MIDAS and opening of the ligand binding site (51).

Structural and mutational studies have investigated models of integrin activation to explore whether integrin extension or leg separation occurs first. Mutations and deletions of the CD-loop (β-subunit terminal domain) have been proposed to keep integrins from extending and have shown no impact on α_V_β_3_ and α_IIb_β_3_ activation (52); there is little proof that mutations in this region affects β_2_ integrins (53), strongly indicating that releasing these constraints is not enough to induce activation.

Structural studies (54) have demonstrated that α_X_β_2_ follows the “switch-blade” model of activation, where leg separation occurs first, releasing constraints of the bent conformation and opening of the ligand-binding site resulting in an intermediate/low affinity conformation E^+^H^−^ (55). The E^+^H^−^ conformation has a free energy between 1.6 and 0.5 kcal/mol, meaning the high affinity conformation is thermodynamically favored (50, 56). Mutations in the EGF3 repeat of the β_2_-subunit have also been shown to induce a high affinity conformation through destabilizing the thermodynamically favorable bent conformation and facilitating leg separation (57). It is noteworthy that an E^−^H^+^ conformation has been described for α_L_β_2_ and α_M_β_2_, allowing integrins to bind ICAM in *cis*, which may regulate neutrophil function (58); however, the specifics of how this activation takes place remain unknown.

Integrin activity is regulated by changes in affinity and aggregation, with the latter affecting receptor avidity. Cytoplasmic proteins bind to α- or β-subunits causing tail separation, stabilizing their high affinity conformation (48, 59). This can be triggered either through signaling from other receptors (“inside-out” signaling, Figure 1B), direct ligand-binding, or experimentally, using Mn^2+^ (“outside-in” signaling, Figure 1C), which triggers downstream signaling pathways (60).

## Inside-Out Signaling

### Rap1 as a Signaling Node

Early studies in complement-dependent phagocytosis using mutants of small GTPases pointed to Rap1 as the main regulator of α_M_β_2_ activity (61) and to it being required for β_1_-mediated phagocytosis (62). Rap1 acted as a node, connecting different signaling pathways (chemokines, fMLP, PAF, and TNFα) for integrin activation (63). Rap1-GTP loading is induced by specific Guanine–Nucleotide Exchange Factors (GEFs), being Epac1 (dependent on cyclic AMP; cAMP) and CalDAG-GEFs (dependent on Ca^2+^/Diacylglycerol; DAG), amongst the best characterized (Figure 1B). Epac1 expression was found to increase during monocyte-macrophage differentiation, correlating with the acquisition of immunoregulatory functions (64), and in neutrophilic HL-60 cells, pharmacological activation of Epac1 increased Rap1-GTP and complement-dependent phagocytosis (65). RasGRP3/CalDAG-GEFIII exhibited similar effects, promoting Rap1 activation and phagocytosis (66). Mutations in CalDAG-GEF1 produced leukocyte adhesion deficiency syndrome (LADIII) with defective neutrophil-endothelial adhesion (67), and mouse CalDAG-GEF1^−^/^−^ macrophages showed reduced integrin activation (68). Rap1 activation can be induced by Toll-like receptors (TLRs) (69); however, the signaling pathways remain poorly defined. In neutrophils, secreted myeloid-related proteins (MRPs) 8 and 14 bind to TLR4 causing Rap1 activation and β_2_-dependent adhesion (70). In macrophages, low concentrations of TLR3/4/9 agonists induced RasGRP3-dependent Rap1 activation (71). Activation of α_M_β_2_ by TLR2/TLR4 required Rac1-GTP loading, PI3K activity, and cytohesin-1 binding to the β_2_ subunit (72).The role of cytohesin-1 is controversial, as the use of cytohesin-1 siRNAs and inhibitors results in an increase in the α_M_β_2_ affinity conformation (73).

### Talin1 and Kindlin-3

Talin1 and Kindlin-3 are the best-characterized integrin activators. Both belong to the FERM family but interact with distinct NPXY motifs in the cytoplasmic tails of β_1_, β_2_, and β_3_, and they thus contribute differently to activation (74). Although Talin-binding is required for efficient β_5_ activation during adhesion, it is dispensable for phagocytosis (75). α_V_β_5_ requires an unknown mediator that recognizes a YEMAS motif proximal to the NPXY. A candidate could be the FERM family FRMD5, as it promotes β_5_-Kindlin-2 interaction and induces ROCK activation during adhesion (76), yet there is no information of its relevance in phagocytosis.

Talin1 contains an N-terminal globular head with a linear FERM domain and a C-terminal rod domain organized in 13 subdomains (R1-R13), which contains a dimerization domain, an integrin binding site, three F-actin binding sites, and several Vinculin and RIAM binding sites (77, 78). The FERM domain has four subdomains (F0-F1-F2-F3), where F3 contains the primary integrin-binding site (IBS) that interacts with the membrane-proximal NPXY motif conserved in β-integrin tails (59, 79, 80). In resting leukocytes, Talin1 remains auto-inhibited due to an interaction between F2F3 and R9 subdomains, which mask the primary IBS (81). Several Talin1 activation mechanisms have been proposed. By binding to PIP5Kγ, Talin1 is recruited to the plasma membrane where the F2F3 domain binds to phosphatidylinositol-4,5-bisphosphate (PI(4,5)P_2_), disrupting the head–tail interaction and exposing the IBS (82, 83). Additionally, RIAM–Talin1 interaction was described as necessary for Talin1 activation and recruitment to integrin tails (Figure 1B) (84).

Hematopoietic cell-specific Kindlin-3 is mutated in LADIII, causing β_1_/β_2_/β_3_ activation defects (85, 86) and preventing neutrophils adhesion to iC3b and ICAM-1 (87). Kindlin-3 binds to the membrane-distal NPKF sequence in the β_2_ subunit tail without excluding Talin1 binding (Figure 1B) (87). Studies of their individual contributions to activation revealed that Kindlin-3 is not sufficient to induce the high-affinity state of α_L_β_2_, whereas Talin1 promotes full activation (88). Whether binding of Talin1 and Kindlin-3 is sequential or simultaneous and their exact contribution to integrin activation remains to be explored. The signaling events directing Kindlin-3 to integrins also remain elusive, as in T cells, Kindlin-3 localization at immune synapses depends on Rap1 and Mst-1/RapL signaling (89), whereas no such interaction has been described for phagocytic cells.

### RIAM–Talin1 Interaction

RIAM (Rap1-Interacting Adaptor Molecule or APBB1IP) was identified as a Rap1 effector that promoted a β_2_ and β_1_ high affinity state, increasing T-cell adhesion and spreading (90). RIAM binds to Rap1-GTP through a central Ras-association domain (RA), to PI(4,5)P_2_ through a Pleckstrin-Homology (PH) domain and to VASP, Profilin, and PLCγ1 via proline-rich regions (90–94). RIAM also interacts with Talin1 through its N-terminus and Talin1 has several RIAM-binding sites located at F3, R2, R3, R8, and R11 subdomains (77). Binding of RIAM to Talin1 releases Talin1 from its autoinhibition (Figure 1B) (95).

The Rap1-RIAM-Talin1-Integrin pathway also operates in complement-dependent phagocytosis. Studies in Talin1-silenced THP-1 cells revealed that Rap1 and Talin1 regulated each other's localization at phagocytic cups (96). Reduced RIAM expression in human monocyte-derived macrophages (MDM), neutrophilic HL-60 cells, and THP-1 cells diminished levels of high affinity α_M_β_2_ and reduced complement-dependent phagocytosis and Talin1 recruitment to phagocytic cups (65). Complement-dependent phagocytosis, cell adhesion to ICAM, and ROS production were also impaired in mouse RIAM^−/−^ macrophages and neutrophils (97). Additionally, RIAM deficiency *in vivo* had a profound effect on β_2_ activity but a moderate effect on β_1_- or β_3_-dependent functions (98).

Besides RIAM, Rap1 effectors RapL and RGS14 (Regulator of G-Protein Signalling-14) have been proposed to regulate α_M_β_2_ activation by inside-out signaling (Figure 1B). The former is proposed to interact with α_M_-subunit inducing integrin tail separation and integrin activation (99); however, RapL has only been shown to interact with a GFFKR motif in α_L_ cytoplasmic tail, and there is no direct evidence that it plays a role in α_M_β_2_ activation (100). For RGS14, the integrin activation mechanism is unknown but seems to be dependent on Talin1-binding to β_2_ (101).

Recently, a direct interaction between Rap1-GTP and Talin1 was described at Talin1 F0 and F1 subdomains (102–105). Synergistic interaction between this region and an F1 lipid-interacting helix facilitates relocation of Talin1 and its integrin-activating function (Figure 1B) (105, 106). This pathway could be relevant for fast cell responses, as disruption in mice impaired platelet aggregation, neutrophil adhesion, extravasation, and phagocytosis but had no effect on macrophage adhesion and migration (104).

## Outside-In Signaling

Outside-in signaling during phagocytosis initiates upon ligand interaction, stabilizing the active conformation, separating integrin tails, allowing for the binding of actin cytoskeletal linkers (Talin1 and/or Kindlin-3), and reorganizing cytoskeletal constraints, as described in the picket-fence model (2). This generates the force needed to drive membrane extension and particle engulfment/internalization (Figure 1C). Regulators have been described in focal complex-like formations at the phagocytic cup (107).

## Clustering and Tyrosine Kinases

One of the earliest events in outside-in signaling could be ligand-induced clustering, a process requiring Talin1 and/or Kindlin-3 (74, 108). Kindlin-3-induced clustering is reported to activate Src family kinases (SFKs) (109, 110) by the exclusion of tyrosine phosphatases such as CD45 (68). Size exclusion of these membrane-bound phosphatases with large extracellular domains seems to be a common feature of integrin-mediated close-contact immune processes, such as Dectin-1 and FcγRIII phagocytosis and immune synapse formation (68, 111, 112). This process does not exclude SFKs but favors their activation due to removing the inhibitory effect of these phosphatases (109, 110). However, there are as of yet only indirect evidences (109, 110) that phosphatases such as CD45 are excluded during integrin-mediated phagocytosis.

SFKs appear to be exclusively involved in “outside-in” signaling, as SFK-deficient cells produced reduced ROS after integrin clustering (113), whereas ICAM-1 adhesion and complement-dependent phagocytosis were normal in pre-activated SFK-deficient cells (114, 115).

A requirement for SFK activation has been described for β_1_, β_2_, and β_3_ integrins (109, 114, 116). Hck, Fgr, and Lyn are the representative SFKs in myeloid cells. Hck co-localized with α_M_β_2_ at phagocytic cups of complement-opsonized zymosan (117, 118), and the Hck knockout phenocopied the α_M_ knockout (119). However, in U937 macrophage-like cells, Hck and Fgr siRNA, unlike Lyn, had no effect on particle internalization (120), and genetic restitution of Fgr-deficient cells inhibited adhesion, spreading, and Syk activation (121). In contrast, the *Hck*^−/−^ *Fgr*^−/−^ *Lyn*^−/−^ triple knockout showed no inhibition in CR3-mediated phagocytosis (122), which may point to compensatory roles of other ubiquitously expressed SFKs. Despite the research into outside-in activation of SFKs, the exact mechanism and individual contribution of each SFK have yet to be dissected.

SFK activity precedes activation of tyrosine kinases Syk and FAK family member Pyk2. Syk is necessary for phagocytosis of iC3b-opsonized beads/zymosan and localizes at phagocytic cups (107, 123), whereas Pyk2 contributes to clearance of complement-opsonized bacteria (124). Clustering of β_2_ integrins results in Syk activation (125), which in turn triggers Pyk2 signaling (126). Pharmacological inhibition of Syk and FAK kinases points to non-redundant functions during phagocytosis and to a possible sequential activation (107).

## Phosphoinositides Coordinate GTPases and Cytoskeletal Rearrangements

Phagocytosis requires sequential enrichment of phosphoinositides (PIPs) in the inner leaflet of the plasma membrane (127). PIP enrichment recruits GEFs for small GTPases, which are sequentially activated (128), and other components of integrin adhesion complexes.

PI(4,5)P_2_ enrichment can be induced by lipid redistribution due to particle-induced plasma membrane deformation (129) and/or by SFK or Talin1-induced PIP5Kγ activity (83, 130, 131). PI(4,5)P_2_ enrichment strengthens Talin1 anchoring (81) and recruits different factors involved in F-actin dynamics, like the actin-depolymerizing-factor ADF/Cofilin, whose activity is inhibited by PI(4,5)P_2_ (132), or the formin mDia (133, 134). Additionally, RIAM binds PI(4,5)P_2_ and may recruit VASP and Profilin, which could also contribute to actin polymerization (90, 93) (Figure 1C).

PI(3,4)P_2_ recruits and induces Vinculin activation through disrupting an auto-inhibitory interaction (135). This is dependent on Syk activity and, to a lesser extent, on FAK/Pyk2 and is upstream from ROCK activation (107). In focal complexes, RIAM contributes to Vinculin binding to Talin1, as RIAM-Talin1 interaction unmasks a Vinculin binding site in Talin1 (77). Afterwards, Vinculin binding to F-actin and α-actinin favors filament bundling and force generation (136, 137).

Increased PI(3,4,5)P_3_ at CR3-phagocytic cups (138) depends on PI3K (139) and Syk (126), and both are activated downstream of Kindlin-induced clustering (140). PI(3,4,5)P_3_ enrichment recruits Vav1/3, which are GEFs for the RhoA family GTPases (128). Complement-dependent phagocytosis requires Vav1 to activate RhoA (61, 141) but also RhoG with no participation from Cdc42 and Rac1 (142). However, expression of constitutively active Rac1 rescues the defective engulfment of Vav1-3 knockouts (143). This discrepancy could be explained by the overlapping roles of RhoG and Rac1 (144, 145) (Figure 1C).

In the final steps leading to engulfment, RhoA-GTP initiates the ROCK-MLCK-myosin signaling pathway and actomyosin contractility (146). RhoA is enriched at phagocytic cups, and its localization is modulated by motifs in β_2_-integrin tails (141). Premature activation of RhoA is inhibited by Rap-GTP through ARAP3, a dual GAP for Rho and Arf GTPases, which is recruited by PI(3,4,5)P_3_ and PI(3,5)P_2_ (147). Finally, mDia contributes to phagosome closure (107, 133) and particle engulfment by connecting the actin cytoskeleton to microtubules (148) (Figure 1C).

## Signaling During Phagocytosis of Apoptotic Cells

During apoptotic cell phagocytosis by mammalian α_V_β_5_/α_V_β_3_, a p130Cas-CrkII-Dock180-Elmo module induces Rac1 activation, which is responsible for cytoskeletal remodeling and phagosome formation (149, 150). Other known signals include the activation of SFKs, as signals from the Mer-TK receptor recruit phosphorylated FAK to mammalian β_5_ in a Src-dependent manner (151), and Syk and Pyk2 activation has been shown to occur for α_V_β_3_ (152, 153). There is also evidence that Rac-1 activation is dependent on RhoG and its GEF Trio (154, 155), whereas RhoA inhibits engulfment (156), and the role of Cdc42 remains unclear (157–159).

An orthologous pathway using the CED-2-CED-5-CED10 module has been described for *C. elegans* INA-1, which activates the Rac ortholog and requires activation of SRC-1(Src-ortholog) (3). Similarly in *Drosophila*, severed axon clearance requires Src42A and Shark—the Src and Syk orthologs, respectively (160, 161)—pointing to an evolutionarily conserved pathway operating in apoptotic cell removal.

## Discussion and Future Perspectives

There are still critical gaps in the knowledge of phagocytic integrin signaling, specifically concerning proximal events and their hierarchy. There are several proposed alternative Talin1-recruitment mechanisms, but their contributions and significance are yet to be established. Rap1-Talin1 interaction is evolutionarily conserved and might constitute a mechanism for short-term adhesions (105), whereas Rap1-RIAM-Talin1 contacts would have a faster recruitment of effector proteins. In this line, it is yet to be established if RIAM is required for outside-in signaling, formation, and recycling of the focal adhesion-like complexes distributed in phagocytic cups (107).

Different F-actin nucleators/elongators are described to participate in CR3-mediated phagocytosis; however, their localization, recruitment, and relative contributions are unknown. The regulation of small GTPases, which control actin dynamics, remains obscure; there is scarce evidence of GEF and GAP spatiotemporal localization in phagocytic cups, and it is well established that GTPases negatively regulate each other, which also raises questions on signal termination and negative-feedback loops.

Many structural and signaling proteins required for phagocytic integrin function have potential post-translational modification-dependent functions, and, although there are several candidates, little work has been undertaken to establish Ser/Thr kinase and phosphatase recruitment and localization within the phagocytic cup.

Fine-grain elucidation of the molecular mechanisms involved in integrin-mediated phagocytosis will yield invaluable information on possible control points for phagocyte functions (antigenic capture, pathogen, tumor or apoptotic body elimination, etc.). Indeed, complement-opsonized immune complexes and particles may be presented directly by subcapsular sinus macrophages to naïve B cells or conveyed to dendritic cells for B-cell presentation. This process requires cooperation between antigen-presenting cell α_M_β_2_/α_X_β_2_ and B-cell CR1, CR2, and/or Fc receptors (162–165). Manipulation of this pathway may inform new vaccine strategies (166).

## Author Contributions

AT-G and EL wrote the original draft. AT-G prepared the figures. Final writing and editing were performed by AT-G, CC, and EL.

## Conflict of Interest

The authors declare that the research was conducted in the absence of any commercial or financial relationships that could be construed as a potential conflict of interest.
